# Cross-Sectional Associations of Intakes of Starch and Sugars with Depressive Symptoms in Young and Middle-Aged Japanese Women: Three-Generation Study of Women on Diets and Health

**DOI:** 10.3390/nu14122400

**Published:** 2022-06-09

**Authors:** Aya Fujiwara, Kentaro Murakami, Hitomi Suga, Satoshi Sasaki, Three-Generation Study of Women on Diets and Health Study Group

**Affiliations:** 1 Department of Epidemiology and Prevention, Center for Clinical Sciences, National Center for Global Health and Medicine, Tokyo 162-8655, Japan; fujiwaraay-tky@umin.ac.jp; 2 Department of Social and Preventive Epidemiology, School of Public Health, University of Tokyo, Tokyo 113-0033, Japan; kenmrkm@m.u-tokyo.ac.jp (K.M.); hitomis-tky@umin.ac.jp (H.S.); 3 Department of Epidemiology and Shokuiku, National Institutes of Biomedical Innovation, Health and Nutrition, Tokyo 162-8636, Japan

**Keywords:** starch, sugars, depressive symptoms, Japanese women, Three-Generation Study of Women on Diets and Health

## Abstract

The relationship between the intakes of saccharide subtypes and depressive symptoms is unclear in Asian countries. This cross-sectional study aimed to investigate this association among 3963 young (age of 18 years) and 3826 middle-aged (mean age of 47.8 years) Japanese women. The intakes of starch, total sugars, free sugars, sucrose, lactose, glucose, and total fructose were assessed using a validated diet history questionnaire. The prevalence of depressive symptoms was 22.0% and 16.8% among young and middle-aged women, assessed using the Center for Epidemiologic Studies Depression (CES-D) score. After adjusting for potential confounding factors, higher starch intake was associated with a lower prevalence of depressive symptoms in young women with an odds ratio (OR) of the fourth to the first quintiles of 0.75 (95% confidence interval (CI): 0.57, 0.99). Moreover, higher intakes of sugars (except for lactose) were associated with a higher prevalence of depressive symptoms in young women, with ORs (95% CI) of the fifth to the first quintiles ranging from 1.30 (0.995, 1.69) for glucose to 1.47 (1.12, 1.93) for sucrose. These associations were not observed in middle-aged women. Future prospective studies are needed to confirm these findings.

## 1. Introduction

Depressive disorder is a major leading cause of the global burden of disease [1]. It affects nearly 280 million people, with an increase greater than 60% in the last three decades, and is more prevalent in women [2]. Moreover, it is estimated that depressive disorder will be the first leading contributor to disease burden worldwide by 2030 [3]. Although the mechanisms underlying the pathogenesis of depression remain unclear, evidence suggests the importance of diet as a crucial factor in the high prevalence of mental disorders and in the prevention of depression [4].

There is a possible relationship between the high consumption of starch and sugars, which are potential dietary factors, and the risk of depressive disorder [5]. Further, this relationship could be mediated mainly by elevated blood glucose level and subsequent insulin secretion. As reported, high intakes of starch and sugars can lead to hypoglycaemia because of an acute response to insulin; thus, this could induce central dysfunction and depressive disorder by influencing hormone levels [6]. Moreover, high intakes of starch and sugars and subsequent insulin secretion could increase the levels of circulating inflammatory markers, which may be associated with the risk of depression [7,8,9,10]. Circulating inflammatory markers reduce the expression of serum brain-derived neurotrophic factor [11], the lower level of which is suggested to reduce neurogenesis and lead to hippocampal atrophy in depression [12]. In contrast, high fructose intake could increase depressive-like behaviour by stimulating the hypothalamic–pituitary–adrenal axis with subsequent elevations in basal corticosterone concentration, independent of blood glucose and insulin [13].

Epidemiological studies have exclusively investigated total and/or added sugars, showing controversial associations with depressive symptoms. Two cross-sectional studies on US adults reported positive associations between total sugars intake and depressive symptoms [14,15]. Other cross-sectional studies on Australian elderly people [16] and Korean adults [17] failed to show any associations, although when considering young women, a negative association was reported in the Korean study [17]. For added sugars, positive associations were reported in cross-sectional studies on Chinese first-year students (estimating total sugars from soft drinks) [18] and US adults [14], as well as a prospective study on Spanish adults [19]. Total sugars intake from sweet foods/beverages was not associated with depressive symptoms but with recurrent depression after five years, in another cross-sectional study on UK adults [20]. However, only one prospective study on US menopausal women focused on the differences in saccharide subtypes and showed positive associations for added sugars, sucrose, and glucose; negative associations for lactose; and no associations for starch, total sugars, and fructose [21].

To the best of our knowledge, no studies of Asian countries have investigated the association with depressive symptoms focusing on differences in saccharide subtypes. The Japanese population has nearly equal levels of starch and reduced sugars intake compared with those of Western countries [22]. Thus, the association between the intake of sugars, not starch, and depressive symptoms may differ between Japan and Western countries. Hence, this study aimed to investigate the association of the intakes of starch and six types of sugars (total sugars, free sugars, sucrose, lactose, glucose, and total fructose) with depressive symptoms in young and middle-aged Japanese women.

## 2. Materials and Methods

### 2.1. Survey Design and Analytic Sample

This cross-sectional study was based on data obtained from the Three-generation Study of Women on Diets and Health, the details of which have been published elsewhere [23,24,25,26]. Briefly, a total of 7016 Japanese students on a dietetic course from 85 higher education institutions (i.e. universities and junior or technical colleges) in 35 of 47 prefectures in Japan were asked to complete two questionnaires on dietary habits and other lifestyle factors during their orientation sessions or first lectures designed for first-year students in April 2011 or 2012. They were also asked to directly share the questionnaires with their mothers and grandmothers or female acquaintances aged 65–89 years and to invite them to participate in the survey. In total, 4933 students (including 4656 women and 277 men; response rate: 70.3%), 4044 mothers (response rate: 57.6%), and 2332 women from the grandmothers’ generation (response rate: 33.2%) completed both questionnaires. The present study was conducted according to the guidelines of the Declaration of Helsinki, and all procedures involving human subjects were approved by the Ethics Committee of University of Tokyo, Faculty of Medicine (No. 3249). Written informed consent was obtained from each participant and for those aged < 20 years also from their parents.

We only included female students aged 18 years (*n* = 4065) to ensure that the young women participants were restricted to first-year students. For middle-aged women, defined according to the most developed world, those aged ≤ 65 years (*n* = 4034) were included [27]. Additionally, since the common age at retirement in Japan is 65 years, the lifestyle of participants aged ≤ 65 and >65 years could differ. We excluded the data from the grandmothers’ generation from the analysis mainly because of the low response rate and the use of different dietary assessment methods. Participants that were excluded from the analysis included: students who completed the questionnaires more than one month after the start of their course, to minimize the influence of dietetic education (*n* = 56); those living in eastern Japan who had participated in the 2011 data collection (because it was assumed that they could not have reported their usual dietary habits and lifestyle due to the occurrence of the Great East Japan Earthquake in March 2011 (*n* = 39 and 63 young and middle-aged women, respectively)); and those from one institution with an extremely low response rate (2%; *n* = 2 for both young and middle-aged women). We also excluded participants with missing information on variables of interest (*n* = 5 and 146 young and middle-aged women, respectively). Finally, the sample sizes were 3963 and 3826 young and middle-aged women, respectively.

### 2.2. Assessment of Depressive Symptoms

Depressive symptoms were assessed using the Japanese version [28] of the Center for Epidemiologic Studies Depression (CES-D) scale [29] included in the lifestyle questionnaire. This scale consists of 20 questions that address six symptoms of depression (depressed mood, guilt or worthlessness, helplessness or hopelessness, psychomotor retardation, loss of appetite, and sleep disturbance) experienced during the preceding week. Each question was scored from 0 to 3 according to the frequency of symptoms, and the total CES-D score ranged from 0 to 60. The criterion validity of the CES-D scale is well established in Western [29] and Japanese [28] participants. Depressive symptoms were defined as present when participants had a CES-D score ≥23 for young women and ≥19 for middle-aged women [25,30], because symptoms overrating has been previously reported in the Japanese [30,31], particularly in younger people [32,33].

### 2.3. Assessment of Intakes of Starch and Sugars

Dietary intake was estimated by using a comprehensive diet history questionnaire (DHQ). The details of the structure, calculation method, and relative validity of commonly consumed foods and nutritional factors have been described elsewhere [34,35,36,37,38,39,40]. Responses to the DHQ and lifestyle questionnaire were checked by the survey staff at the study centre. In the event of any missing or erroneous responses to questions considered essential for the analysis, the participants were asked to provide a response to the question again.

Energy intake (EI) was calculated using an ad hoc computer algorithm for the DHQ based on the Standard Tables of Food Composition in Japan (STFCJ), 2010 [41]. The intakes of starch and six types of sugars were calculated based on a recently developed starch and sugars composition database for Japanese populations [22]. Total sugars were defined as the sum of all monosaccharides (i.e., glucose, fructose, and galactose) and disaccharides (sucrose, lactose, maltose, and trehalose) [42]. Free sugars were defined as sugars added by the manufacturer, cook, or consumer and as sugars naturally present in honey, syrups, fruit juices, and fruit juice concentrates [43]. Fructose was calculated as the total fructose, i.e., the sum of free fructose and 50% of sucrose [44,45]. The DHQ has a satisfactory ranking ability of the energy-adjusted intakes of starch and sugars in Japanese women [46]; Spearman’s correlation coefficients between the DHQ and 16-day dietary record ranged from 0.45 (starch) to 0.58 (total sugars) in the residual method [47].

### 2.4. Assessment of Covariates

A diet quality score (ranging from 0 to 70) was calculated based on seven food components (i.e. grain dishes; vegetable dishes; fish and meat dishes; milk; fruits; energy from snacks, confectioneries and beverages; and Na from seasonings) according to previously developed algorithms [24,25] using the dietary intakes estimated with the DHQ. This score was calculated because it has previously shown association with depressive symptoms in the present population [25].

The body mass index (BMI) was calculated as self-reported body weight (kg) divided by the square of the height (m). Information on current smoking status (yes or no), current alcohol drinking status (yes or no), medication use (yes or no), and stress level (very low, low, normal, high, or very high) was used. Physical activity was calculated as the total metabolic equivalent (hours per day), based on the frequency and duration of seven activities (walking, cycling, standing, running, high-intensity activities, sleeping, and sedentary activity) over the preceding month [48,49]. Dietary reporting status (plausible, under-, and over-reporters) was evaluated on the basis of the ratio of the reported EI to the basal metabolic rate (BMR) using Goldberg’s cut-off [50], as described elsewhere [24]. In addition, living alone was considered for young women. In contrast, age (calculated from the date of birth), current marital status (married or unmarried), education (≤12, 13–14, or ≥15 years), and occupation (housewife, part-time job, or full-time job) were considered for middle-aged women.

### 2.5. Statistical Analysis

All statistical analyses were conducted separately for young and middle-aged women using SAS statistical software (version 9.4; SAS Institute Inc., Cary, NC, USA). All the reported *p*-values were two-tailed, and statistically significance was set at *p* < 0.05. Descriptive data are presented as means and standard deviations (SDs) for continuous variables and as numbers and percentages for categorical variables. To minimise the influence of dietary misreporting [51,52], the intakes of starch and sugars, as well as the diet quality score, were energy-adjusted using the residual method [47]. We calculated the mean value of the contribution of each food group to the intakes of starch and sugars. The food groups were defined based on culinary usage and nutrient profile similarities, mainly according to the STFCJ [41]. Pearson’s correlation coefficients between the intakes of starch and sugars were calculated to examine the association between these saccharides. The energy-adjusted intakes of starch and sugars were categorised at quintile points based on the distribution of each generation. Associations between the selected variables and the intakes of starch and total sugars were examined using a linear trend test (for continuous variables) and the Mantel–Haenszel chi-squared test (for categorical variables). Odds ratios (ORs) and 95% confidence intervals (CIs) for depressive symptoms were estimated for each quintile category of the intakes of starch and sugars using logistic regression analyses. The lowest quintile category of the intakes of starch and sugars was used as the reference category. Three models were considered for analysis. Model 1 was a crude model. In Model 2, the following non-dietary factors were adjusted: BMI, current smoking status, alcohol drinking status, medication use, self-reported level of stress, physical activity, and dietary reporting status (for both age groups); living alone (for young women); age, marital status, education, and occupation (for middle-aged women). In Model 3, further adjustments were made for the diet quality score. We tested for linear trends with increasing levels of the intakes of starch and sugars by assigning to each participant a median value for the category as a continuous variable.

## 3. Results

### 3.1. Intakes of Starch and Sugars and Their Sources

All the young women were 18 years old, while the mean (SD) age of middle-aged women was 47.8 (4.1) years. The prevalence of depressive symptoms was 22.0% (*n* = 871) for young women and 16.8% (*n* = 643) for middle-aged women. For young women, the mean energy-adjusted intakes (SD) were 149.8 (38.5) g/d for starch and 65.2 (25.4) g/d for total sugars (Table 1). The corresponding values for middle-aged women were 151.6 (39.1) g/d and 64.6 (21.5) g/d, respectively. In both age groups, the major food sources were rice, grains and confectioneries for starch; sugar and confectioneries for total sugars, free sugars, sucrose, and total fructose; dairy products and confectioneries for lactose; and vegetables, fruits, and sugar-sweetened beverages for glucose. Sugar-sweetened beverages were also the main contributors to the intakes of total and free sugars and total fructose in young women. In middle-aged women, fruits were the main contributors to the intakes of total sugars and total fructose, and dairy products were the main contributors to total sugars intake.

Starch intake was negatively correlated with each sugar intake. In contrast, the intakes of sugars were positively associated with each other, except for lactose and glucose in young women (Table 2).

### 3.2. Associations of Intakes of Starch and Sugars with Selected Characteristics

Selected characteristics according to the quintiles of the intakes of starch and total sugars are shown in Table 3 (for young women) and Table 4 (for middle-aged women). Participants in the highest quintile of starch intake more likely had a higher BMI, were non-drinkers and medication non-users, under-reported the EI, and had higher diet quality scores. Furthermore, these participants more likely lived alone, had a higher level of stress, and were physically inactive among young women, but they were more likely older among middle-aged women. Similarly, participants in the highest quintile of the intakes of sugars were more likely older and non-drinkers (middle-aged women only) and under-reported EI (young women only). In contrast, these participants more likely used medications and had a lower level of stress (for both age groups); lower BMI and were physically active (young women only); or had a higher education status (middle-aged women only). In addition, while total sugars intake was negatively associated with the diet quality score in young women, positive associations were observed in middle-aged women. Because of the positive correlations between total sugars and other sugars, the distribution of these characteristics according to the quintiles of the intakes of other sugars was similar to the intake of total sugars (data not shown).

### 3.3. Associations of Intakes of Starch and Sugars with Depressive Symptoms

Table 5 shows the associations of the intakes of starch and sugars with depressive symptoms in young women. After adjusting for non-dietary and dietary covariates (Model 3), the OR (95% CI) for depressive symptoms of the fourth to the first quintiles of starch intake was 0.75 (0.57, 0.99), while the OR (95% CI) of the fifth quintile was 0.84 (0.65, 1.10) (*p* for trend = 0.15). Conversely, fully adjusted ORs (95% CI) for depressive symptoms of the fifth to the first quintiles were 1.42 (1.09, 1.86) for total sugars; 1.42 (1.09, 1.88) for free sugars; 1.47 (1.12, 1.93) for sucrose; 1.30 (0.995, 1.69) for glucose; and 1.38 (1.05, 1.86) for total fructose (all *p* for trend ≤ 0.01). In middle-aged women, no significant associations were observed between intakes of starch and sugars and depressive symptoms after adjusting for all covariates investigated (Model 3), except for sucrose, which had an OR (95% CI) of the second to the first quintiles of 0.73 (0.53, 0.99) (*p* for trend = 0.50) (Table 6).

## 4. Discussion

In this cross-sectional study, starch intake was negatively associated with depressive symptoms in young Japanese women, whereas the intakes of sugars (except for lactose) were positively associated. However, these associations were not observed in middle-aged women. To our knowledge, this is the first study to investigate the intakes of the subtypes of saccharides in relation to depressive symptoms in non-Western populations with relatively low intakes of sugars.

The mean energy-adjusted intakes of starch and sugars were similar between the two age groups in this study. Although these intakes were comparable with those in previous Japanese [22] and Korean [17] studies, the present population of Japanese women had a higher starch intake and lower intakes of sugars than the Western population [16,19,21].

In line with previous cross-sectional [14,15] and prospective [18,19,20,21] studies, we found positive associations of total sugars, free sugars, sucrose, and glucose intakes with depressive symptoms in the present study, at least for young Japanese women. Meanwhile, the associations of starch intake (negative), lactose (no significant associations), and total fructose (positive) with depressive symptoms in the present population of young Japanese women differed from those in the US prospective study on menopausal women with no significant associations for starch and fructose and a negative association for lactose [21]. The associations of total and free sugars, sucrose, and glucose intakes with depressive symptoms could be a result of insulin secretion induced by an elevated blood glucose level and subsequent changes in hormone [6] and circulating inflammatory marker [7,8,9,10] levels, as well as the expression of serum brain-derived neurotrophic factor [11,12]. For total fructose, the association with depressive symptoms could be due to the increase in basal corticosterone concentration via hypothalamic–pituitary–adrenal axis stimulation [13]. The higher starch and (total) fructose intakes and the lower lactose intake in the young Japanese women in this study may partly explain the difference in the associations compared with the previous US study on menopausal women [21]. However, we cannot rule out the possibility that the observed associations (including the absence of associations) of each saccharide intake in these young Japanese women reflect the correlations between the intakes of these saccharides and free sugars.

Although the intakes of starch and sugars were comparable between young and middle-aged women in the present study, we did not observe any associations with depressive symptoms in middle-aged women. Previous cross-sectional [16,17,20] and prospective studies [21], which also reported no associations of total sugars and sugars from sweet foods/beverages with depressive symptoms, were conducted on middle-aged and elderly individuals. Therefore, the difference between the considered young and middle-aged women may be due to healthier dietary habits in older individuals [53]. For example, previous studies have hypothesised that the null association between total sugars intake and depressive symptoms was because of the food sources with higher fibre content, which slows down carbohydrate metabolism [16,21]. In the present study, the food sources of the intakes of starch and sugars were generally similar in both age groups. However, sugar-sweetened beverages were the main contributors to the intakes of total and free sugars and total fructose in young women, whereas fruits and dairy products were the main contributors to total sugars and total fructose (fruits only) in middle-aged women. These differences may cancel the adverse effects of the intakes of sugars on depressive symptoms in middle-aged women.

Another explanation is the difference in stress-coping strategies between the age groups. Young women more often eat something to cope with stress than their older counterparts [31]. Thus, the intakes of sugars could be changed by the influence of the mental status, especially in young women (i.e., reverse causality) [54]. We confirm that the analysis excluding participants whose dietary habits changed within the previous year (*n* = 593 young women and *n* = 349 middle-aged women) did not alter the present associations (data not shown). Nevertheless, the cross-sectional design of the study did not permit the assessment of causality because of the uncertain temporality of the assessment. The relationship between dietary intake and mental health is complex and likely to be bidirectional. The temporal direction of the association between diet quality and depressive symptoms could go both ways [55]. Nevertheless, only a prospective study would provide a better understanding of the relationship between the intakes of starch and sugars and depressive symptoms in Japan and Western countries [20,56].

The present study has several limitations. First, the educational level of the participants in this study (i.e., dietetic students and their mothers) may be relatively high, because only 56.2% of Japanese adolescents currently undergo studies in colleges or universities [57]. Furthermore, compared with the general population, dietetic students are likely to be more conscious of their diet, although in most institutions, the present survey was conducted within one month after the start of the dietetic course to minimise the effect of dietetic education. Hence, the present results might not be generalisable to the general Japanese population. Second, we assessed depressive symptoms using the CES-D, a validated questionnaire [28,29], rather than structured diagnostic interviews. The absence of a clinical diagnosis may have led to the inclusion of participants with chronic fatigue syndrome or atypical depression. Third, all self-reported dietary assessment methods are subject to random and systematic measurement errors. To minimise these errors, we assessed dietary habits during the preceding month using the DHQ, a well-established dietary assessment questionnaire. Although the DHQ is not specifically designed to measure the intakes of starch and sugars, it has satisfactory validity regarding the intakes of starch and sugars against a 16-day weighed dietary record, as described above. Additionally, we used energy-adjusted dietary intakes [51,52] and adjusted for the dietary reporting status. Fifth, we did not assess the sample size for the present analysis because the Three-generation Study of Women on Diets and Health Study was designed to investigate relationships between various exposures and outcomes among different generations of women. The present study, however, has sufficient power to detect the association of the intakes of starch and sugars with depressive symptoms with statistical significance. Finally, although we adjusted for various potential confounding variables, including both dietary and non-dietary variables, residual confounding may remain.

## 5. Conclusions

In this study, negative association of starch intake and positive associations of sugars intakes (except for lactose) with depressive symptoms were observed in young Japanese women but not in middle-aged women, with an equivalent intake of starch and a reduced intake of sugars compared with those in Western countries. Further research, especially using a prospective design, should be conducted to confirm the present findings.

## Figures and Tables

**Table 1:** Contribution of each food group to the intakes of starch and sugars in 3963 young and 3826 middle-aged Japanese women ^1^.

	Young Women							Middle-Aged Women						
	Starch	Total Sugars	Free Sugars	Sucrose	Lactose	Glucose	Total Fructose	Starch	Total Sugars	Free Sugars	Sucrose	Lactose	Glucose	Total Fructose
Intakes (g/day) ^2^														
Mean	149.8	65.2	45.0	36.5	6.9	9.9	27.6	151.6	64.6	40.6	38.4	7.2	8.8	27.0
Standard deviation	38.5	25.4	23.4	13.8	5.7	7.1	12.4	39.1	21.5	17.9	13.6	5.5	4.8	10.1
Contribution (%)														
Rice and grains	62.9	0.8	0	0.3	0	4.2	0.2	60.2	0.7	0	0.2	0	4.1	0.2
Bread	9.3	2.3	0	<0.1	1.9	3.6	1.9	10.4	2.4	0	<0.1	2.1	4.2	2.0
Noodle	9.8	0.4	0	0.1	0	0	0.1	9.9	0.4	0	0.1	0	0	0.1
Other grain products	3.1	0.6	0.6	0.4	0.1	0.8	0.6	2.8	0.5	0.5	0.3	0.1	0.7	0.5
Potatoes	2.9	0.4	0	0.4	0	0.8	0.5	3.1	0.5	0	0.5	0	0.9	0.5
Sugar and confectioneries	10.3	49.1	67.4	71.3	0	11.7	18.4	11.8	48.8	73.2	70.5	0	10.3	23.6
Pulses and nuts	0.3	3.1	2.7	3.4	0	6.4	2.5	0.3	3.8	3.6	4.2	0	7.4	3.3
Vegetables ^3^	0.4	7.1	0.3	2.0	0	21.0	8.1	0.5	8.3	0.4	2.3	0	26.5	9.6
Fruits	0.3	7.6	0.3	6.4	0	11.7	9.8	0.4	10.2	0.2	8.9	0	16.1	13.0
Fish and shellfish	0.2	0.9	1.3	1.4	0	0.5	0.95	0.2	1.4	2.3	2.1	0	0.8	1.53
Meats	<0.1	0.3	0.4	0.2	0	0.7	0.1	<0.1	0.2	0.4	0.2	0	0.7	0.1
Eggs	0	0.2	0	0	0	1.5	0	0	0.2	0	0	0	1.4	0
Dairy products	<0.1	9.2	4.0	3.9	47.8	0.7	2.7	<0.1	10.5	4.1	3.7	59.0	0.7	2.6
Fat and oil	<0.1	0.6	0.8	0.9	0.1	0.4	0.64	<0.1	0.5	0.7	0.7	0.1	0.4	0.51
Tea and coffee	0	0	0	0	45.9	0	33.0	0	0	0	0	35.7	0	29.5
Sugar-sweetened beverages ^4^	<0.1	10.7	14.2	6.0	4.2	21.3	12.0	<0.1	6.7	9.6	4.5	3.0	12.5	7.6
Fruit and vegetable juice	0.1	4.8	6.3	1.9	0	8.7	6.6	<0.1	2.6	3.6	0.9	0	5.3	3.6
Alcoholic beverages	0	<0.1	0	0	0	<0.1	<0.1	0	0.1	0	0	0	0.6	0.1
Seasonings	<0.1	1.8	1.4	0.8	0	5.8	1.5	<0.1	2.0	1.5	0.8	0	7.3	1.5

^1^ All values are means unless otherwise indicated. ^2^ Energy-adjusted using the residual method. ^3^ Including mushrooms and seaweeds. ^4^ Consisting of soda, sports drinks, fruit drinks, and milk beverages.

**Table 2:** Pearson’s correlation coefficients among the intakes of starch and sugars in 3963 young (above diagonal) and 3826 middle-aged (below diagonal) Japanese women ^1,2^.

	Starch	Total Sugars	Free Sugars	Sucrose	Lactose	Glucose	Total Fructose
Starch	---	−0.55	−0.45	−0.51	−0.31	−0.31	−0.49
Total sugars	−0.42	---	0.93	0.82	0.31	0.78	0.97
Free sugars	−0.34	0.87	---	0.78	0.09	0.79	0.94
Sucrose	−0.37	0.86	0.84	---	0.16	0.36	0.79
Lactose	−0.27	0.39	0.11	0.17	---	−0.02	0.08
Glucose	−0.24	0.70	0.61	0.35	0.03	---	0.83
Total fructose	−0.25	0.70	0.59	0.35	0.14	0.94	---

^1^ Energy-adjusted values (g/d) obtained using the residual model were used. ^2^ All correlations were significantly different from zero (*p* < 0.05), except for that between lactose and glucose for young women (*p* = 0.12).

**Table 3:** Selected characteristics of 3963 young Japanese women according to the quintiles of the energy-adjusted intakes of starch and total sugars ^1,2^.

	Starch ^3^							Total Sugars ^3^						
	Q1 (*n* = 792)		Q3 (*n* = 793)		Q5 (*n* = 792)		*p* ^4^	Q1 (*n* = 792)		Q3 (*n* = 793)		Q5 (*n* = 792)		*p* ^4^
BMI (kg/m^2^), mean ± SD	20.6	2.6	20.9	2.9	21.0	3.0	0.007	21.2	3.2	20.9	2.6	20.5	2.7	<0.001
Currently smoking, *n* (%)														
Yes	3	(0.4)	0	(0.0)	2	(0.3)	-	2	(0.3)	1	(0.1)	3	(0.4)	-
Alcohol drinking, *n* (%)														
Yes	68	(8.6)	44	(5.6)	43	(5.4)	<0.001	46	(5.8)	46	(5.8)	62	(7.8)	0.06
Medication use, *n* (%)														
Yes	105	(13.3)	86	(10.8)	73	(9.2)	0.003	84	(10.6)	72	(9.1)	108	(13.6)	0.003
Living alone, *n* (%)														
Yes	111	(14.0)	193	(24.3)	244	(30.8)	<0.001	121	(21.6)	208	(26.2)	166	(21.0)	0.26
Self-reported level of stress, *n* (%)														
Very low	32	(4.0)	24	(3.0)	15	(1.9)	0.01	17	(2.2)	18	(2.3)	39	(4.9)	<0.001
Low	204	(25.8)	174	(21.9)	198	(25.0)		189	(23.9)	188	(23.7)	225	(28.4)	
Normal	456	(57.6)	481	(60.7)	471	(59.5)		480	(60.6)	463	(58.4)	437	(55.2)	
High	62	(7.8)	81	(10.2)	62	(7.8)		70	(8.8)	80	(10.1)	68	(8.6)	
Very high	38	(4.8)	33	(4.2)	46	(5.8)		36	(4.6)	44	(5.6)	23	(2.9)	
Dietary reporting status, *n* (%) ^5^														
Under-reporting	126	(15.9)	140	(17.7)	147	(18.6)	<0.001	50	(6.3)	205	(25.9)	145	(18.3)	<0.001
Plausible reporting	553	(69.8)	632	(79.7)	571	(72.1)		636	(80.3)	568	(71.6)	558	(70.5)	
Over-reporting	113	(14.3)	21	(2.7)	74	(9.3)		106	(13.4)	20	(2.5)	89	(11.2)	
Physical activity (total metabolic equivalents; h/d), mean ± SD	38.7	6.1	37.6	5.3	37.6	5.5	<0.001	37.7	5.3	37.4	5.5	38.5	6.0	<0.001
Energy intake (kcal/d), mean ± SD	1938	761	1654	425	1795	632	<0.001	2010	609	1567	418	1852	737	<0.001
Diet quality score, mean ± SD ^3,6^	39.5	8.2	41.3	7.8	41.8	7.3	<0.001	41.9	6.6	41.0	8.1	40.0	8.3	<0.001

Q, quintile; SD, standard deviation. ^1^ The values in the second and fourth quintiles are not shown for simplicity. ^2^ All participants were 18 years old. ^3^ Energy-adjusted using the residual method. ^4^ For continuous variables, a linear trend test was used with the median value in each quintile category of the energy-adjusted intakes of starch (108.7, 133.7, 148.8, 164.0, and 187.1 g/day) and total sugars (41.1, 54.0, 63.4, 72.8, and 90.3 g/day) as a continuous variable in linear regression; for categorical variables, a Mantel–Haenszel chi-squared test was used. ^5^ Under-reporting was defined as a ratio of reported energy intake (EI) to basal metabolic rate (BMR) < 1.09; plausible reporting as an EI: BMR of 1.09–2.21; and over-reporting as an EI: BMR > 2.21. ^6^ The diet quality score (0–70) was calculated using the intakes of ‘grain dishes’, ‘vegetable dishes’, ‘fish and meat dishes’, ‘milk’, ‘fruits’, energy from ‘snacks, confectioneries and beverages’, and Na from seasonings.

**Table 4:** Selected characteristics of 3826 middle-aged Japanese women according to the quintiles of the energy-adjusted intakes of starch and total sugars ^1^.

	Starch ^2^							Total Sugars ^2^						
	Q1 (*n* = 765)		Q3 (*n* = 766)		Q5 (*n* = 765)		*p* ^3^	Q1 (*n* = 765)		Q3 (*n* = 766)		Q5 (*n* = 765)		*p* ^3^
Age (years), mean ± SD	47.5	4.1	47.6	4.2	48.1	4.1	0.002	47.1	3.9	48.1	3.9	48.2	4.2	<0.001
BMI (kg/m^2^), mean ± SD	22.0	3.0	21.9	3.1	22.3	3.3	0.04	22.3	3.3	22.1	3.1	21.9	3.1	0.10
Currently smoking, *n* (%)														
Yes	94	(12.3)	61	(8.0)	43	(5.6)	<0.001	76	(9.9)	49	(6.4)	57	(7.5)	0.06
Alcohol drinking, *n* (%)														
Yes	473	(61.8)	371	(48.4)	276	(36.1)	<0.001	477	(62.4)	363	(47.4)	310	(40.5)	<0.001
Medication use, *n* (%)														
Yes	225	(29.4)	181	(23.6)	168	(22.0)	0.002	172	(22.5)	208	(27.2)	248	(32.4)	<0.001
Marital status, *n* (%)														
Married	686	(89.7)	708	(92.4)	688	(89.9)	0.62	705	(92.2)	711	(92.8)	688	(89.9)	0.07
Education, *n* (%)														
≤12 years	373	(48.8)	353	(46.1)	407	(53.2)	0.23	404	(52.8)	343	(44.8)	369	(48.2)	0.02
13 to 14 years	281	(36.7)	300	(39.2)	260	(34.0)		275	(36.0)	304	(39.7)	277	(36.2)	
≥15 years	111	(14.5)	113	(14.8)	98	(12.8)		86	(11.2)	119	(15.5)	119	(15.6)	
Occupation, *n* (%)														
Housewife	145	(19.0)	138	(18.0)	153	(20.0)	0.53	122	(16.0)	165	(21.5)	145	(19.0)	0.27
Part-time job	350	(45.8)	339	(44.3)	335	(43.8)		368	(48.1)	323	(42.2)	347	(45.4)	
Full-time job	270	(35.3)	289	(37.7)	277	(36.2)		275	(36.0)	278	(36.3)	273	(35.7)	
Self-reported level of stress, *n* (%)														
Very low	48	(6.3)	45	(5.9)	56	(7.3)	0.13	52	(6.8)	42	(5.5)	62	(8.1)	0.046
Low	227	(29.7)	225	(29.4)	212	(27.7)		199	(26.0)	238	(31.1)	233	(30.5)	
Normal	404	(52.8)	436	(56.9)	434	(56.7)		439	(57.4)	417	(54.4)	407	(53.2)	
High	55	(7.2)	37	(4.8)	40	(5.2)		52	(6.8)	41	(5.4)	39	(5.1)	
Very high	31	(4.1)	23	(3.0)	23	(3.0)		23	(3.0)	28	(3.7)	24	(3.1)	
Dietary reporting status, *n* (%) ^4^														
Under-reporting	45	(5.9)	51	(6.7)	77	(10.1)	<0.001	33	(4.3)	75	(9.8)	37	(4.8)	0.33
Plausible reporting	531	(69.4)	672	(87.7)	585	(76.5)		607	(79.4)	648	(84.6)	593	(77.5)	
Over-reporting	189	(24.7)	43	(5.6)	103	(13.5)		125	(16.3)	43	(5.6)	135	(17.7)	
Physical activity (total metabolic equivalents; h/day), mean ± SD	41.1	5.6	40.6	5.5	40.5	5.9	0.06	40.7	5.8	40.8	5.5	41.1	5.7	0.17
Energy intake (kcal/d), mean ± SD	2112	587	1761	379	1817	708	<0.001	1994	654	1716	428	2004	552	0.02
Diet quality score, mean ± SD ^2,5^	40.3	8.4	43.0	8.0	44.2	7.2	<0.001	40.5	7.4	43.3	8.1	43.2	8.6	<0.001

Q, quintile; SD, standard deviation. ^1^ The values in the second and fourth quintiles are not shown for simplicity. ^2^ Energy-adjusted using the residual method. ^3^ For continuous variables, a linear trend test was used with the median value in each quintile category of the energy-adjusted intakes of starch (110.6, 135.8. 151.2, 166.2, and 187.3 g/day) and total sugars (42.5, 54.4, 62.7, 71.8, and 89.1 g/day) as a continuous variable in linear regression; for categorical variables, a Mantel–Haenszel chi-squared test was used. ^4^ Under-reporting was defined as a ratio of reported energy intake (EI) to basal metabolic rate (BMR) < 1.09; plausible reporting as an EI: BMR of 1.09–2.21; and over-reporting as an EI: BMR > 2.21. ^5^ The diet quality score (0–70) was calculated using the intakes of grain dishes; vegetable dishes; fish and meat dishes; milk; fruits; energy from snacks, confectioneries, and beverages; and Na from seasonings.

**Table 5:** Odds ratios (95% confidence intervals) for depressive symptoms according to the quintiles of the energy-adjusted intakes of starch and sugars in 3963 young Japanese women.

	Q1 (*n* = 792)		Q2 (*n* = 793)			Q3 (*n* = 793)			Q4 (*n* = 793)			Q5 (*n* = 792)			*p* for Trend ^1^
Starch (g/day), median ^2^	108.7		133.7			148.8			164.0			187.1			
Depressive symptoms (%) ^3^	25.3		22.3			22.6			18.9			20.8			
Model 1 ^4^	1.00	(reference)	0.85	(0.68,	1.07)	0.86	(0.69,	1.09)	0.69	(0.54,	0.88)	0.78	(0.62,	0.99)	0.01
Model 2 ^5^	1.00	(reference)	0.83	(0.63,	1.07)	0.98	(0.75,	1.27)	0.72	(0.55,	0.94)	0.80	(0.62,	1.05)	0.06
Model 3 ^6^	1.00	(reference)	0.84	(0.65,	1.10)	1.01	(0.77,	1.31)	0.75	(0.57,	0.99)	0.88	(0.65,	1.10)	0.15
Total sugars (g/day), median ^2^	41.1		54.0			63.4			72.8			90.3			
Depressive symptoms (%) ^3^	19.4		20.4			19.2			22.7			28.2			
Model 1 ^4^	1.00	(reference)	1.06	(0.83,	1.36)	0.98	(0.77,	1.26)	1.22	(0.96,	1.55)	1.62	(1.29,	2.05)	<0.001
Model 2 ^5^	1.00	(reference)	1.15	(0.87,	1.52)	1.02	(0.77,	1.36)	1.24	(0.94,	1.63)	1.49	(1.14,	1.94)	0.003
Model 3 ^6^	1.00	(reference)	1.14	(0.87,	1.51)	1.01	(0.76,	1.34)	1.21	(0.92,	1.59)	1.42	(1.09,	1.86)	0.008
Free sugars (g/day), median ^2^	23.8		34.9			42.7			50.9			66.5			
Depressive symptoms (%) ^3^	18.9		18.3			21.2			22.8			28.7			
Model 1 ^4^	1.00	(reference)	0.96	(0.74,	1.23)	1.15	(0.90,	1.47)	1.27	(0.99,	1.61)	1.72	(1.36,	2.18)	<0.001
Model 2 ^5^	1.00	(reference)	1.04	(0.78,	1.38)	1.31	(0.99,	1.73)	1.33	(1.01,	1.76)	1.61	(1.24,	2.11)	<0.001
Model 3 ^6^	1.00	(reference)	1.00	(0.75,	1.33)	1.23	(0.93,	1.63)	1.20	(0.90,	1.60)	1.42	(1.07,	1.88)	0.006
Sucrose (g/day), median ^2^	22.5		30.1			35.4			41.1			51.6			
Depressive symptoms (%) ^3^	19.3		18.4			20.7			22.3			29.2			
Model 1 ^4^	1.00	(reference)	0.94	(0.73,	1.21)	1.09	(0.85,	1.39)	1.20	(0.94,	1.53)	1.72	(1.36,	2.17)	<0.001
Model 2 ^5^	1.00	(reference)	1.05	(0.79,	1.39)	1.27	(0.96,	1.68)	1.23	(0.93,	1.63)	1.65	(1.27,	2.14)	<0.001
Model 3 ^6^	1.00	(reference)	1.03	(0.78,	1.37)	1.21	(0.91,	1.60)	1.13	(0.85,	1.49)	1.47	(1.12,	1.93)	0.004
Lactose (g/day), median ^2^	2.1		4.1			5.6			7.9			12.6			
Depressive symptoms (%) ^3^	22.2		20.9			22.3			20.6			23.9			
Model 1 ^4^	1.00	(reference)	0.93	(0.73,	1.18)	1.01	(0.79,	1.27)	0.91	(0.71,	1.15)	1.10	(0.87,	1.39)	0.37
Model 2 ^5^	1.00	(reference)	0.96	(0.73,	1.26)	1.07	(0.81,	1.41)	0.97	(0.74,	1.27)	1.06	(0.81,	1.38)	0.65
Model 3 ^6^	1.00	(reference)	0.96	(0.73,	1.26)	1.07	(0.81,	1.41)	1.04	(0.79,	1.37)	1.19	(0.91,	1.56)	0.14
Glucose (g/day), median ^2^	4.4		7.3			8.9			10.9			15.1			
Depressive symptoms (%) ^3^	22.5		17.9			22.3			20.4			26.8			
Model 1 ^4^	1.00	(reference)	0.75	(0.59,	0.96)	0.99	(0.78,	1.26)	0.89	(0.70,	1.13)	1.26	(1.00,	1.59)	0.009
Model 2 ^5^	1.00	(reference)	0.76	(0.58,	1.01)	0.94	(0.72,	1.24)	0.90	(0.68,	1.19)	1.25	(0.96,	1.63)	0.02
Model 3 ^6^	1.00	(reference)	0.79	(0.60,	1.05)	0.98	(0.74,	1.29)	0.93	(0.71,	1.23)	1.30	(0.995,	1.69)	0.01
Total fructose (g/day), median ^2^	16.5		22.4			26.3			30.9			39.0			
Depressive symptoms (%) ^3^	19.1		20.3			19.3			24.1			27.2			
Model 1 ^4^	1.00	(reference)	1.08	(0.84,	1.39)	1.02	(0.79,	1.30)	1.35	(1.06,	1.71)	1.58	(1.25,	2.00)	<0.001
Model 2 ^5^	1.00	(reference)	1.26	(0.95,	1.66)	1.11	(0.84,	1.47)	1.48	(1.13,	1.95)	1.47	(1.12,	1.92)	0.003
Model 3 ^6^	1.00	(reference)	1.23	(0.93,	1.63)	1.08	(0.81,	1.43)	1.40	(1.06,	1.85)	1.38	(1.05,	1.81)	0.01

Q, Quintile. ^1^ Logistic regression models were used with the median value in each quintile category of the intakes of starch and sugars as a continuous variable in logistic regression. ^2^ Energy-adjusted using the residual method. ^3^ Depressive symptoms were defined as present when subjects had a Center for Epidemiologic Studies Depression score ≥ 23. ^4^ Crude model. ^5^ Adjustment was made for body mass index (kg/m^2^; continuous), current smoking status (yes or no), alcohol drinking status (yes or no), medication use (yes or no), living alone (yes or no), self-reported level of stress (very low, low, normal, high, or very high), dietary reporting status (under-reporting, plausible reporting, or over-reporting), and physical activity (total metabolic equivalent; h/day; continuous). ^6^ Adjustment was made for variables in Model 2 plus energy-adjusted diet quality score (continuous).

**Table 6:** Odds ratios (95% confidence intervals) for depressive symptoms according to the quintiles of the energy-adjusted intakes of starch and sugars in 3826 middle-aged Japanese women.

	Q1 (*n* = 765)		Q2 (*n* = 765)			Q3 (*n* = 766)			Q4 (*n* = 765)			Q5 (*n* = 765)			*p* for Trend ^1^
Starch (g/day), median ^2^	110.6		135.8			151.2			166.2			187.3			
Depressive symptoms (%) ^3^	17.4		15.6			18.0			14.9			18.2			
Model 1 ^4^	1.00	(reference)	0.88	(0.67,	1.15)	1.04	(0.80,	1.36)	0.83	(0.63,	1.09)	1.06	(0.81,	1.37)	0.84
Model 2 ^5^	1.00	(reference)	0.94	(0.70,	1.27)	1.17	(0.87,	1.57)	0.95	(0.70,	1.28)	1.11	(0.82,	1.49)	0.52
Model 3 ^6^	1.00	(reference)	0.98	(0.73,	1.33)	1.24	(0.92,	1.67)	1.03	(0.76,	1.40)	1.20	(0.89,	1.62)	0.23
Total sugars (g/day), median ^2^	42.5		54.4			62.7			71.8			89.1			
Depressive symptoms (%) ^3^	18.6		13.9			15.0			18.4			18.2			
Model 1 ^4^	1.00	(reference)	0.71	(0.54,	0.93)	0.78	(0.59,	1.02)	0.99	(0.77,	1.28)	0.97	(0.75,	1.26)	0.40
Model 2 ^5^	1.00	(reference)	0.71	(0.53,	0.96)	0.76	(0.57,	1.03)	1.00	(0.75,	1.33)	0.87	(0.65,	1.16)	0.98
Model 3 ^6^	1.00	(reference)	0.75	(0.55,	1.01)	0.80	(0.59,	1.08)	1.07	(0.80,	1.44)	0.90	(0.67,	1.20)	0.82
Free sugars (g/day), median ^2^	23.8		32.0			38.3			45.7			59.8			
Depressive symptoms (%) ^3^	15.8		14.3			16.3			16.7			20.9			
Model 1 ^4^	1.00	(reference)	0.88	(0.67,	1.17)	1.04	(0.79,	1.36)	1.07	(0.82,	1.40)	1.41	(1.09,	1.83)	0.002
Model 2 ^5^	1.00	(reference)	0.84	(0.61,	1.14)	0.99	(0.73,	1.34)	1.04	(0.77,	1.40)	1.16	(0.87,	1.56)	0.10
Model 3 ^6^	1.00	(reference)	0.84	(0.62,	1.14)	0.97	(0.71,	1.31)	0.98	(0.72,	1.32)	1.04	(0.78,	1.40)	0.47
Sucrose (g/day), median ^2^	24.4		31.6			37.0			43.3			54.6			
Depressive symptoms (%) ^3^	17.4		13.3			15.3			18.2			19.9			
Model 1 ^4^	1.00	(reference)	0.73	(0.55,	0.97)	0.86	(0.65,	1.12)	1.06	(0.81,	1.37)	1.18	(0.91,	1.53)	0.02
Model 2 ^5^	1.00	(reference)	0.70	(0.52,	0.95)	0.86	(0.64,	1.16)	1.06	(0.79,	1.42)	1.03	(0.77,	1.38)	0.18
Model 3 ^6^	1.00	(reference)	0.73	(0.53,	0.99)	0.87	(0.64,	1.17)	1.04	(0.77,	1.39)	0.96	(0.72,	1.29)	0.50
Lactose (g/day), median ^2^	2.1		4.2			6.0			8.8			13.3			
Depressive symptoms (%) ^3^	19.6		16.6			14.2			15.8			17.8			
Model 1 ^4^	1.00	(reference)	0.82	(0.63,	1.06)	0.68	(0.52,	0.89)	0.77	(0.59,	1.00)	0.89	(0.69,	1.15)	0.59
Model 2 ^5^	1.00	(reference)	0.81	(0.61,	1.08)	0.71	(0.52,	0.95)	0.80	(0.60,	1.08)	0.90	(0.67,	1.19)	0.75
Model 3 ^6^	1.00	(reference)	0.83	(0.62,	1.11)	0.77	(0.57,	1.05)	0.96	(0.71,	1.31)	1.13	(0.83,	1.53)	0.17
Glucose (g/day), median ^2^	4.9		6.7			8.0			9.7			13.3			
Depressive symptoms (%) ^3^	19.2		16.9			16.5			14.9			16.6			
Model 1 ^4^	1.00	(reference)	0.85	(0.66,	1.11)	0.83	(0.64,	1.08)	0.74	(0.56,	0.96)	0.84	(0.64,	1.09)	0.17
Model 2 ^5^	1.00	(reference)	0.84	(0.63,	1.12)	0.92	(0.68,	1.23)	0.74	(0.55,	0.99)	0.77	(0.58,	1.03)	0.07
Model 3 ^6^	1.00	(reference)	0.87	(0.65,	1.16)	0.98	(0.73,	1.32)	0.81	(0.60,	1.09)	0.86	(0.64,	1.15)	0.30
Total fructose (g/day), median ^2^	17.1		22.2			25.9			30.2			38.5			
Depressive symptoms (%) ^3^	17.8		15.2			15.7			18.2			17.3			
Model 1 ^4^	1.00	(reference)	0.83	(0.63,	1.08)	0.86	(0.66,	1.12)	1.03	(0.79,	1.33)	0.96	(0.74,	1.26)	0.68
Model 2 ^5^	1.00	(reference)	0.84	(0.63,	1.14)	0.84	(0.63,	1.13)	1.02	(0.76,	1.37)	0.84	(0.62,	1.12)	0.53
Model 3 ^6^	1.00	(reference)	0.86	(0.64,	1.17)	0.86	(0.63,	1.15)	1.04	(0.78,	1.40)	0.84	(0.63,	1.13)	0.54

Q, Quintile. ^1^ Logistic regression models were used with the median value in each quintile category of intakes of starch and sugars as a continuous variable in logistic regression. ^2^ Energy-adjusted using the residual method. ^3^ Depressive symptoms were defined as present when subjects had a Center for Epidemiologic Studies Depression score ≥ 19. ^4^ Crude model. ^5^ Adjustment was made for body mass index (kg/m^2^; continuous), current smoking status (yes or no), alcohol drinking status (yes or no), medication use (yes or no), marital status (married or unmarried), educational level (≤12, 13–14, ≥15 years), occupation (housewife, part-time job, or full-time job), self-reported level of stress (very low, low, normal, high, or very high), dietary reporting status (under-reporting, plausible reporting, or over-reporting), and physical activity (total metabolic equivalent; h/day; continuous). ^6^ Adjustment was made for variables in Model 2 plus energy-adjusted diet quality score (continuous).

## Data Availability

The datasets generated and analysed during the present study are not publicly available due to privacy and ethical restrictions imposed by the Ethics Committee of University of Tokyo, Faculty of Medicine, but are available from the corresponding author upon reasonable request. The questionnaires used in this study are available from the corresponding author upon reasonable request.

## Supplementary Materials

The following supporting information can be downloaded at: https://www.mdpi.com/article/10.3390/nu14122400/s1.
