# Supplementary material for: Cross-Sectional Associations of Intakes of Starch and Sugars with Depressive Symptoms in Young and Middle-Aged Japanese Women: Three-Generation Study of Women on Diets and Health
Source: Nutrients. 2022 Jun 9;14(12):2400. doi: 10.3390/nu14122400 (PMC9229452; doi:10.3390/nu14122400)
Supplement: Supplementary file 1 [file nutrients-14-02400-s001.zip › nutrients-1755339-supplementary.pdf]

Three-generation Study of Women on Diets and Health Study Group Members:

Natsuko Sato-Mito, Eka Fujimoto, Hiroyuki Nakamura, Shirou Awata, Takashi Hoshino, Tomoko Watanabe, Ayuho Suzuki, Keiko Mori, Yoshie Manabe, Hideto Matsuda, Tomoko Imai, Megumi Murakami, Tomoko Koda, Miho Kogirima, Kazumi Kikuchi, Ayako Nemoto, Hitomi Hayabuchi, Yasumi Kimura, Momoe Iwami, Hiroko Tsuchiya, Kimiko Nishimura, Katsuhiko Minamino, Mihoko Murata, Hiroko Kito, Ritsuko Tanaka, Tetsuko Tejima, Hiromi Tanaka, Yoshie Niizawa, Keiko Miyamaru, Yuriko Kamitsubo, Shoko Miyata, Hiroaki Kanouchi, Rumiko Shimizu, Masafumi Saito, Yumiko Hori, Kei Nakajima, Kazuyo Tsugita, Minami Murakawa, Hiromi Watanabe, Kazuhiro Uenishi, Tomoko Shindo, Noriko Oyama, Emi Arimura, Mikako Yamashita, Keizo Kodama, Tomoyuki Sumizawa, Takiko Sagara, Kanazawa Gakuin, Yoko Komatsu, Fusako Teramoto, Kazuya Kitamori, Satomi Maruyama, Toshiyuki Kohri, Naoko Kaba, Yasuyo Asano, Kumiko Asahi, Tosei Takahashi, Yoshiko Kasahara, Harumi Hirata, Yukiko Misumi, Ryoko Yamamura, Hiroko Yamashita, Tomoe Matsunaga, Naomi Yamaguchi, Shiho Miura, Shiho Miyazaki, Yuji Mizuno, Masaki Miyake, Makiko Kaneko, Chiemi Tokudome, Naoko Hirota, Naoko Okishima, Naoko Mizuno, Kimiko Miyahara, Kazumi Dokai, Tamami Oyama, Yumi Yoshioka, Hiroko Nakazawa, Akiko Sato, Tomiko Tsuji, Yukiko Okami, Hiroko Tsuda, Katsumi Imai, Ririko Moriguchi, Keiko Shinohara, Nobuko Murayama, Osamu Kushida, Kayoko Sawano, Noriko Horita, Kenichiro Yasutake, Megumi Kubota, Kazue Nakata, Yuko Higashine, Michinori Kurokawa, Nobuko Taniguchi, Hiroko Hashimoto, Hisae Mori, Aki Takagi, Sakiko Aoyama, Miyoko Honda, Shoko Komatsu, Yasuhiko Iwase, Sayo Uesugi, Minatsu Kobayashi, Reiko Hikosaka, Hiroko Moriwaki, Hisanori Minami, Nana Nakashima, Satomi Ishii, Sumiko Yasukawa, Yuri Kintaka, Mieko Aoki, Chieko Teraoka, Namie Tsumura, Mai Nakata, Shinji Ikemoto, Natsu Hiroki, Naoko Kawano, Kiyomi Osawa, Ikuyo Kawakami, Kazuko Ohki, Seigo Shiga, Hidemichi Ebisawa, Yumi Funahashi, Tomomi Nagahata, Yukiko Mita, Junko Tsudzuki, Kazuko Yoshizawa, Masaaki Tanaka, Sonoko Ayabe, Mieko Kanbe, Chihiro Hirakata, Junko Suzuki, Kanae Sato, Katsumi Shibata, Tsutomu Fukuwatari, Mitsue Sano, Yoko Ichikawa, Midori Morooka, Nozomi Saito, Ryoko Nishiyama, Hiroyuki Tanaka, Jun Oka, Noriko Sekiguchi, Ryoko Wada, Terue Shioiri, Akiko Notsu, Sumiko Harada, Akira Ohmori, Toyomi Kuwamori, Masako Ota, Tomohiro Yano, Ayami Sato, Miki Sekine, Chisato Abe, Reiko Watanabe, Kanako Muramatsu, Eiji Takeda, Hisami Yamanaka-Okumura, Chisaki Adachi, Yoshiko Takahashi, Yuri Yaguchi, Kumiko Suizu, Mayumi Shigeta, Sanae Fukasawa, Masako Fujii, Akiko Sakuma, Keisuke Arao, Tetsuko Kato
